# DFT Evaluation of Metal Ion Selectivity in Protein Phosphatase PPM1A: The Effect of Native Metal Type and Multiplicity on the Competition with Other Biogenic Contenders for the Active Site

**DOI:** 10.3390/biom16060860

**Published:** 2026-06-11

**Authors:** Nikoleta Kircheva, Vladislava Petkova, Silvia Angelova, Todor Dudev

**Affiliations:** 1 Institute of Optical Materials and Technologies “Acad. J. Malinowski”, Bulgarian Academy of Sciences, 1113 Sofia, Bulgaria; nkircheva@iomt.bas.bg (N.K.); vpetkova@iomt.bas.bg (V.P.); sea@iomt.bas.bg (S.A.); 2 University Centre on Tautomeric Research and Education in Science and Technology (ERA Chair UCTREST), University of Plovdiv, 4000 Plovdiv, Bulgaria; 3 Faculty of Chemistry and Pharmacy, Sofia University “St. Kliment Ohridski”, 1164 Sofia, Bulgaria

**Keywords:** protein phosphatase PPM1A, metal ion selectivity, metal ion competition, density functional theory

## Abstract

Protein phosphatase PPM1A plays a critical role in cellular signaling by dephosphorylating key regulatory proteins. According to experimental data, the enzyme requires either Mn^2+^ or Mg^2+^ bound in the active center(s), hence its catalytic activity strongly depends on the chelated metal ions. In this study, the metal ion selectivity of PPM1A is investigated using DFT calculations on active site constructs of bi- and trinuclear metal centers and protein ligands from the first and second metal coordination shells. Binuclear Mn-Mn and trinuclear Mn-Mn-Mn sites show poor resistance to substitution by biogenic Fe^2+^ and Zn^2+^, with Gibbs energies of the Mn^2+^ → Fe^2+^/Zn^2+^ exchange being consistently negative in both the gas phase and condensed media. In contrast, Mg-Mg and Mg-Mg-Mg centers are substantially more robust, with a thermodynamically unfavorable Mg^2+^ → Fe^2+^/Zn^2+^ substitution—except in the case of the Mg-Mg-Zn complex. The primary factors governing this metal competition in the modeled structures are the nature of the competing cation and the solvation properties of its aqua complexes, while solvent exposure of the binding site and the number of metal cations in the catalytic center exert a comparatively minor effect. Overall, these findings demonstrate that Mg^2+^-loaded active sites offer considerably greater protection against biogenic metal displacement than their Mn^2+^ counterparts, thus shedding light on the metalloprotein stability and enzyme fidelity of PPM1A.

## 1. Introduction

Intricate biochemical processes in the body such as cellular metabolism, signal transduction, and gene regulation are strictly governed by enzymes [1,2]. For the proper functioning of about one third of those biomolecules, 24 various types of metal cofactors have been found to play a crucial role, the most common being Na^+^, K^+^, Mg^2+^, Ca^2+^, Zn^2+^, Mn^2+^, Fe^2+/3+^, Co^2+/3+^, Ni^2+^ and Cu^+/2+^ [3,4,5]. Therefore, metal ions known as ‘biogenic’ or ‘native’ not only grant structural stability, but are also essential for the catalytic activity of the enzyme. The physicochemical properties of the bound metal ion, e.g., ionic radius, preferred coordination geometry, ligand type, and electrostatic interactions within the binding pocket, along with the surrounding environment formed by the coordinating amino acid residues, collectively regulate the proper overall performance of the metal–protein complexes [2]. On the other hand, the presence in the cellular milieu of other cations able to ‘compete’ with the native ones for binding in the specific active sites and thus affecting the enzymatic activity remains an important and intriguing objective in bioinorganic chemistry, which can be studied at an atomistic level with the tools of computational chemistry [6,7,8,9].

A group of enzymes of immense significance comprise the protein phosphatases. They intensely participate in the regulation of protein activity by removing phosphate groups from their substrates via the process of dephosphorylation [10,11]. The current study focuses on the most popular representative of the group of the metal-dependent Ser/Thr protein phosphatase PPM family: PPM1A, also known as PP2Cα. Its crystal structure was first reported by Barford et al. in 1996 [12]. An N-terminal catalytic domain and a C-terminal protein domain collectively taking the form of two β-sandwiches and four α-helices build the molecule of PPM1A, whereas its catalytic center is located on the apex of the β-sandwiches. For the proper functioning of the enzyme, at least two, but sometimes three metal ions, preferably Mg^2+^ and Mn^2+^ cations, are required [10,13,14]. Interestingly, comprehensive analysis with different divalent metals revealed that ferrous ions can also activate PPM phosphatases [15]. The function of the metal cofactor in the binuclear active site is to capture one water molecule to subsequently attack the phosphate groups of the diverse substrates—AMP-activated protein kinase (AMPK), mitogen-activated protein kinase (MAPK), cyclin-dependent kinases (CDK), etc. [16,17,18]. The presence of a third metal ion bound to the Asp146/Asp239 subunit stabilizes the natural structure of the enzyme by limiting the conformational mobility of both the active site and the specific region of the flip subdomain. This theory is confirmed by the loss of activity upon mutation of aspartic to glutamic acid [19,20]. The importance of the amino acid residues chelating the metal ions is further demonstrated by the fact that they are highly conserved in eukaryotes. In binuclear human PPM1A, one metal ion is coordinated to the carbonyl group of glycine (Gly61) and one oxygen of the aspartic acid carboxyl group (Asp60), while the other metal cofactor is bound to the second oxygen of Asp60 along with two additional aspartate residues (Asp239 and Asp282). Other residues conserved near the catalytic domain assist most probably in forming and stabilizing the structure of the catalytic center. Overall, these facts provide strong evidence that PPM1A strictly depends on the bound metal cations and the architecture of its active site, unlike other phosphatase subclasses that diverge in a rather mechanistic way.

Distributed within almost all tissues, PPM1A is localized in both the nucleus and cytoplasm [21]. The immense significance of the protein phosphatase can clearly be seen in the wide variety of biochemical processes that it is crucial for—wound healing, inflammation, neovascularization, regulation of bone morphogenetic protein signaling, formation of the placenta, synthesis of oocytes, differentiation of nerve cells, just to name a few [22,23,24]. Hence, special attention should be paid to PPM1A’s therapeutic potential in diseases such as antiviral immunity, osteoarthritis, fibrotic disorders, and most importantly cancer [25,26,27,28,29,30,31]. For accomplishing this purpose, however, the main principles governing the processes of structural integrity, metal binding and selectivity should first be clarified. A systematic evaluation could be achieved by applying an integrated computational and experimental approach, e.g., relatively time-efficient, but highly reliable DFT calculations based on and in complement to experimental data allow for the evaluation of thermodynamic parameters that regulate metal substitution reactions and shed light on the stability of the PPM1A’s active site. In the present study, we report results based on a previously applied DFT methodology on deciphering the metal competition processes in metalloproteins [32,33,34,35,36] that aim to answer the following puzzling questions in regard to PPM1A from a thermodynamic point of view: (a) how does the number of metal cations (two or three) in the active site of the enzyme affect its metal selectivity; (b) which metal center in the binuclear or trinuclear configuration is more/most susceptible to a single attack from other non-native metal cations; (c) how does the type of the native cation (Mn^2+^ or Mg^2+^) influence the stability of the binding site toward substitution by other non-cognate metal species; (d) how does the degree of solvent exposure of the active site affect the metal substitution process; (e) does, and if so, how does, metal substitution alter the original structure of the metal binding site?

## 2. Methods

### 2.1. Studied Structures

In answering the above questions, we modeled metal binding sites (initial structures taken from the experiment) comprising two (PDB entry 1A6Q, [12]) and three (PDB entry 6B67, [19]) metal cations. Protein ligands and crystalline water molecules from the metals’ first and second coordination shell were retained during the optimizations. Asp^−^/Glu^−^ side chains and backbone peptide fragments were modeled as CH_3_CH_2_COO^−^ and CH_3_CONHCH_3_, respectively. Furthermore, active sites containing either Mn^2+^ or Mg^2+^ were modeled. The competition with other divalent biogenic, but non-native, metals (Fe^2+^ and Zn^2+^) for the metal binding site, based on the free energy of metal substitution reactions, was studied. Each native metal ion position in the active site was probed for replacement by a non-native cation (single metal substitution) and its vulnerability to the metal exchange process was assessed. Free energy in the gas phase was first evaluated, followed by solution calculations (via thermodynamic cycle) at ε = 4 (buried binding sites) and ε = 29 (solvent-accessible binding sites). The SMD solvation energies for the protein–metal complexes at these dielectric media were calculated, whereas the respective metal–aqua complexes were considered to be in a water environment characterized by ε = 78. High-spin electron configuration was considered for the open-shell Mn^2+^ and Fe^2+^ in accordance with experimental and theoretical findings showing that both cations are namely a high-spin configuration in complexes containing weak-field oxygen-donor ligands such as H_2_O, CH_3_COO- and CH_3_CONHCH_3_ [37,38].

### 2.2. Modeled Reactions

The first metal substitution in Mn/Mg binuclear binding sites with Fe or Zn is modeled as:

[Fe/Zn–6wat]^2+^ + [Mn/Mg−Mn/Mg]^−^ → [Fe/Zn−Mn/Mg]^−^ + [Mn/Mg–6wat]^2+^ (R1)

The second metal substitution is analogously modeled: the substitution complex is [Mn/Mg–Fe/Zn]^−^.

The first metal substitution in Mn/Mg trinuclear binding sites with Fe or Zn:

[Fe/Zn–6wat]^2+^ + [Mn/Mg–Mn/Mg–Mn/Mg]^+^ → [Fe/Zn–Mn/Mg–Mn/Mg]^+^ + [Mn/Mg–6wat]^2+^ (R2)

Second and third metal substitutions are analogously modeled (the substitution complexes are [Mn/Mg–Fe/Zn–Mn/Mg]^+^ and [Mn/Mg–Mn/Mg–Fe/Zn]^+^).

### 2.3. Computational Methodology

The software package for quantum-chemical calculations Gaussian 16 was implemented in executing all the required computations [39]. First, a full geometry optimization of the studied constructs necessary for the modeled reactions was performed without imposing any geometrical constraints, followed by vibrational frequency analysis, both at the B3LYP/6-31+G(3d,p) level of theory for all atoms [40]. The latter provided evidence that the studied structure is indeed a local minimum in the potential energy surface, as no imaginary frequencies were observed for any of the geometries. This particular combination of a method/basis set was chosen as it has proven reliable when modeling metal substitution reactions in biomolecules in our previous works (See Table 1 and Table 2 below), but it also reproduced metal–ligand distances in both currently studied bi- and trinuclear active sites in accordance with the deposited experimental data. Average Mn_1/2_–O (ligand) bonds were calculated to be 2.15/2.22 Å as compared with 2.15/2.17 Å in the binuclear center of 1A6Q, whereas the averaged Mg_1/2/3_–O (ligand) distances were calculated to be 2.04/2.11/2.11 Å in the optimized model as opposed to the trinuclear center of 6B67 where the respective metal–O (ligand) distances were found to be 2.24/2.22/2.22 Å but, notably, in a Ca^2+^-chelated complex. Furthermore, the optimization and vibrational frequency analysis provided the essential electronic energies, E_elect_, thermal energies, including the zero-point energy, E_th_, and entropy, S, required for the calculation of the Gibbs energy in the gas phase, ∆G^1^, at room temperature T = 298 K and atmospheric pressure of 1 atm according to the Equation [41]:

∆G^1^ = ∆E_elect_ + ∆E_th_ − T∆S, (1)

The ‘∆’ symbol stands for the respective differences between the products and reagents of Reactions R1 or R2. As mentioned above, the effect of the surrounding medium was further evaluated through the implementation of the thermodynamic cycle, where single-point calculations of the optimized-in-the-gas-phase structures were performed at the SMD level [42]. The solvents of choice characterized by their dielectric constant ε are diethyl ether (ε ≈ 4) and propanonitrile (ε ≈ 29), mimicking a closed-shell active site and a site that is relatively open to the solvent binding pocket, respectively. Note that the metal–aqua complexes were considered to be in aqueous solution of ε = 78. Consequently, the energy of metal substitution in a medium characterized by dielectric constants of ε denoted as upper indices ∆G^4^ and ∆G^29^ were obtained in accordance with Equation (2):

∆G^ε^ = ∆G^1^ + ∆E_solv_^ε^ (products) − ∆E_solv_^ε^ (reagents) (2)

From a thermodynamic point of view, the herewith calculated changes in the Gibbs energies disclose the reactions’ outcome in regards to energetics and spontaneity: a modeled reaction with ∆G < 0 corresponds to a metal binding site prone to metal exchange (a plausible substitution), whereas ∆G > 0 provides evidence for a metal center protected against an outer attack.

The computational methodology applied in the current study has been successfully implemented in numerous studies for determining the outcome of metal competition in various molecular systems. In Table 1, calculated at the B3LYP/6-31+G(3d,p) level of theory, mean metal–ligand bond distances and experimentally reported data are compared.

**Table 1:** Calculated (at B3LYP/6-31+G(3d,p) level of theory) and experimental mean metal–ligand bond distances in Å in various metal-containing molecular systems.

Molecule	Bond	Calculated	Experimental
[Li(H_2_O)_4_]^+^	Li-O	1.95 ^a^	1.94 ± 0.05 ^b^
[Mg(H_2_O)_6_]^2+^	Mg-O	2.10 ^a^	2.07 ± 0.03 ^b^
[Zn(H_2_O)_6_]^2+^	Zn-O	2.12 ^c^	2.08 ^d^
[Zn(acetonitrile)_6_]^2+^	Zn-N	2.16 ^c^	2.12 ^e^
Mn_1_^2+^-PPM1A	Mn_1_-O	2.15 ^f^	2.15 ^g^
Mn_2_^2+^-PPM1A	Mn_2_-O	2.22 ^f^	2.17 ^g^
[Fe(H_2_O)_6_]^3+^	Fe-O	2.05 ^h^	2.00 ± 0.01 ^i^
[Ga(H_2_O)_6_]^3+^	Ga-O	1.96 ^h^	1.96 ± 0.01 ^j^
[Na(18-crown-6)]^+^	Na-O	2.75 ^k^	2.77 ± 0.07 ^l^
[K(18-crown-6)]^+^	K-O	2.82 ^k^	2.80 ± 0.04 ^l^

^a^ from Ref. [32]; ^b^ from Ref. [43]; ^c^ from Ref. [35]; ^d^ from Ref. [44]; ^e^ from Ref. [45]; ^f^ present study; ^g^ from Ref. [12]; ^h^ from Ref. [33]; ^i^ from Ref. [46]; ^j^ from Ref. [47]; ^k^ from Ref. [34]; ^l^ analysis from the Cambridge Structural Database [48].

The applied computational approach is further justified in terms of Gibbs energies as it reproduces well not only qualitatively, but also quantitatively the outcome of metal substitution reactions. The comparison between calculated and experimental ∆G (in kcal/mol) is presented in Table 2.

**Table 2:** Calculated (at B3LYP/6-31+G(3d,p) level of theory) and experimental Gibbs energies (kcal/mol) of metal substitution reactions in various metal-containing molecular systems.

Modeled Reaction	Calc.	Exp.
[K(18-crown-6)]^+^ + [Na(H_2_O)_6_]^+^ → [Na(18-crown-6)]^+^ + [K(H_2_O)_6_]^+^	1.4 ^a^	2.0 ^b^
[Mg(H_2_O)_5_(CH_3_COO^−^)] + [Li(H_2_O)_4_]^+^ → [Mg(H_2_O)_3_(CH_3_COO^−^)] + [Mg(H_2_O)_6_]^2+^	1.3 ^c^	0.8 ^d^
[Mg(H_2_O)_4_(C_2_O_4_^2−^)] + [Li(H_2_O)_4_]^+^ → [Li(H_2_O)_2_(C_2_O_4_^2−^)] + [Mg(H_2_O)_6_]^2+^	2.1 ^a,e^	2.7 ^d^
[Mg(H_2_O)_2_(NTA^3−^)] + [Li(H_2_O)_4_]^+^ → [Li(NTA^3−^)] + [Mg(H_2_O)_6_]^2+^	4.1 ^a,f^	4.1 ^d^
[Zn(EDTA)]^2−^ + [Cu(H_2_O)_6_]^2+^ → [Cu(EDTA)]^2−^ + [Zn(H_2_O)_6_]^2+^	−2.5 ^g^	−3.1 ^h^
[Fe^3+^-transferrin] + [Ga^3+^(H_2_O/OH)_6_] → [Ga^3+^-transferrin] + [Fe^3+^(H_2_O/OH)_6]_	4.0 ^i^	2.9 ^j^

^a^ from Ref. [34]; ^b^ from Ref. [49]; ^c^ from Ref. [32]; ^d^ from Ref. [50]; ^e^ C_2_O_4_^2−^ = oxalate; ^f^ NTA = nitrilotriacetic acid; ^g^ from Ref. [35]; ^h^ from Ref. [51]; ^i^ from Ref. [52]; ^j^ from Ref. [53].

Moreover, single-point calculations of the B3LYP/6-31+G(3d,p) optimized structures either with the triple zeta basis set, or with additional empirical dispersion on the structures required to compute the Zn^2+^ → Mg^2+^ substitution reaction in a binuclear active site yield results with no substantial difference. The Gibbs energies in all modeled media—gas phase, diethyl ether, and propanenitrile—vary from 0.3 to 0.8 kcal/mol when applying the triple-zeta basis set, and from 1.2 to 1.7 kcal/mol with the addition of dispersion. Most importantly, these deviations do not affect the overall observed tendencies of metal substitution.

Although a thorough justification of the applied computational methodology is presented, it should be noted that the current study employs simplified, cluster-based static models representing the first and second coordination spheres surrounding the investigated metal ions in PPM1A. Consequently, the models do not explicitly account for long-range electrostatic interactions, protein flexibility, solvent dynamics, or effects arising beyond the second coordination shell. Therefore, the reported results should be interpreted as local metal-substitution free energies rather than as a complete description of the protein’s free-energy landscape. A more comprehensive treatment would require conformational sampling through QM/MM approaches and molecular dynamics simulations. Nevertheless, despite these limitations, the applied DFT methodology provides consistent comparative insights into the thermodynamic factors governing metal competition and enables reliable trend analysis for the bi- and trinuclear PPM1A active-site models.

## 3. Results

### 3.1. Binuclear Mn-Mn Binding Site

**Figure 1:**
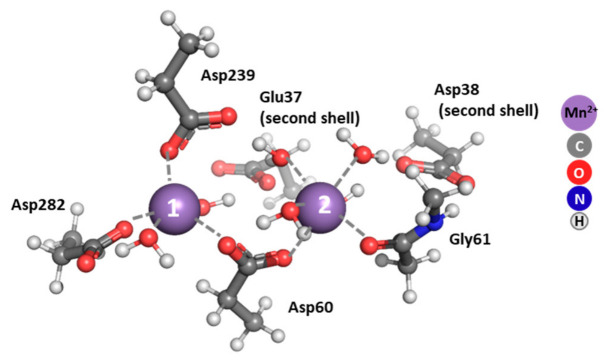
B3LYP/6-31+G(3d,p) fully optimized binuclear Mn^2+^ binding site of PPM1A.

The PPM1A active site comprising two Mn^2+^ cations was modeled after the 1A6Q X-ray structure and subsequently subjected to geometry optimization and frequency analysis. The optimized structure of the metal construct is shown in Figure 1. Metal 1 (Mn_1_^2+^) is directly coordinated to three Asp^−^ side chains (Asp60, Asp239 and Asp282), whereas the second manganese cation (Mn_2_^2+^) is bound to Asp60 and the backbone carbonyl group of Gly61. Asp60 serves as a bridge between the two metal species. The rest of the metals’ first coordination sphere is complemented by water molecules. Other two carboxylate ligands are located in the metals’ second coordination layer. Manganese cations in the optimized Mn^2+^–Mn^2+^ construct were replaced by ferrous cations (single-metal substitutions) and the respective structures were taken as a starting point for further full optimization without imposing any geometrical constraints on the mixed Fe^2+^–Mn^2+^ and Mn^2+^–Fe^2+^ complexes.

**Figure 2:**
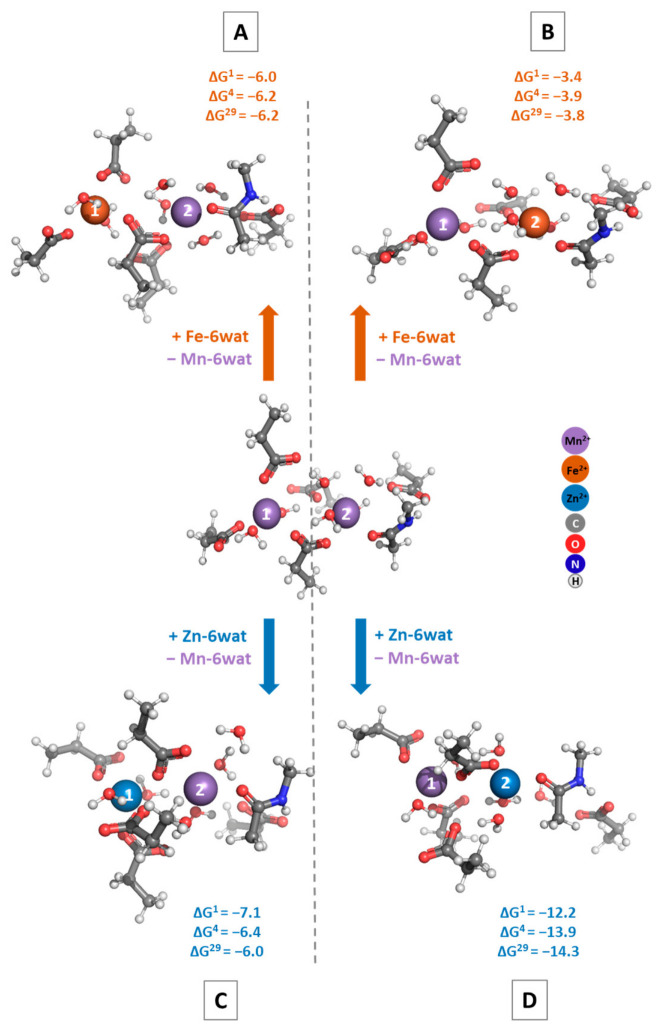
B3LYP/6-31+G(3d,p) fully optimized binuclear metal centers, containing Mn^2+^, Fe^2+^ and Zn^2+^ cations and Gibbs energies of Mn^2+^ → Fe^2+^/Zn^2+^ exchange (in kcal/mol): substitution of Mn_1_^2+^ (**A**) and Mn_2_^2+^ (**B**) cations with Fe^2+^; substitution of Mn_1_^2+^ (**C**) and Mn_2_^2+^ (**D**) cations with Zn^2+^.

Substituting each of the native Mn^2+^ cations with Fe^2+^ does not change the overall shape of the binding site. The respective Fe^2+^-occupied active centers shrink; however, upon Mn^2+^ → Fe^2+^ substitution: The mean Mn_1_^2+^–ligand bond distance of 2.147 Å in the Mn-Mn complex decreases to 2.106 in the Fe^2+^–Mn^2+^ construct. Similarly, the average Mn_2_^2+^–ligand bond length of 2.218 Å shortens to 2.167 in the Mn^2+^–Fe^2+^ structure. Accordingly, the gas phase Gibbs energies for the two metal exchange reactions became negative (meaning favorable Mn^2+^ → Fe^2+^ substitutions) (Figure 2A,B) as the ligand affinity of Fe^2+^ relative to that of Mn^2+^ is higher [54]. It should be noted that condensed-phase calculations reveal the same trend since the solvation Gibbs energies of Mn/Fe aqua and protein complexes are almost equal: ΔG_solv_^78^([Mn/Fe-6wat]^2+^) = −209.9/−209.0 kcal/mol and ΔG_solv_^29^([Mn-Mn/Mn-Fe]^−^) = −50.0/−49.5 kcal/mol and ΔG_solv_^29^([Mn-Mn/Fe-Mn]^−^) = −50.0/−49.3 kcal/mol. Solvent exposure of the binding site is apparently not a major factor determining the metal selectivity in this system as the respective ΔG_solv_^4^ and ΔG_solv_^29^ are not quite different and fluctuate in very narrow limits (Figure 2A,B; see also Figure 2C,D).

The outcome of the competition between Mn^2+^ and Zn^2+^ was studied as well. The Zn^2+^-occupied structures and the respective ΔGs are presented in Figure 2C,D. Zn^2+^ has much higher ligand affinity as compared to Mn^2+^ [54]. Expectedly, the ligand coordination sphere around the newcomer shrinks quite significantly as compared to that of the cognate Mn^2+^: mean values from 2.147 Å to 2.070 Å for Mn_1_^2+^ and Zn_1_^2+^, respectively, and from 2.218 Å to 2.161 Å for Mn_2_^2+^ and Zn_2_^2+^, respectively. Calculations also suggest that Zn^2+^ is a very successful competitor of Mn^2+^ as evidenced by the pretty low Gibbs energies of the Mn^2+^ → Zn^2+^ substitution in both the gas phase and condensed media (Figure 2C,D). The calculations suggest that each of the Mn^2+^ centers is susceptible to alien (Fe^2+^ or Zn^2+^) attack.

### 3.2. Binuclear Mg-Mg Binding Site

**Figure 3:**
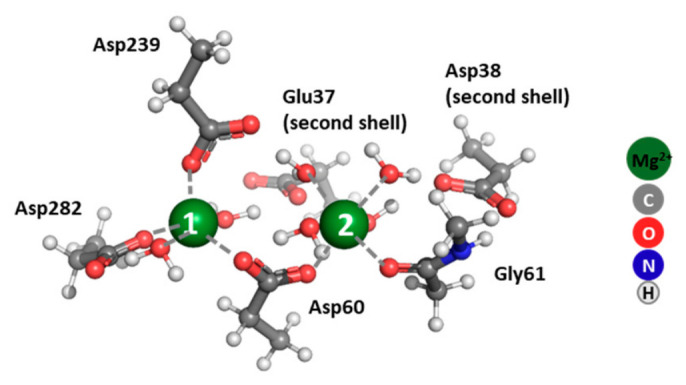
B3LYP/6-31+G(3d,p) fully optimized binuclear Mg^2+^ binding site of PPM1A.

**Figure 4:**
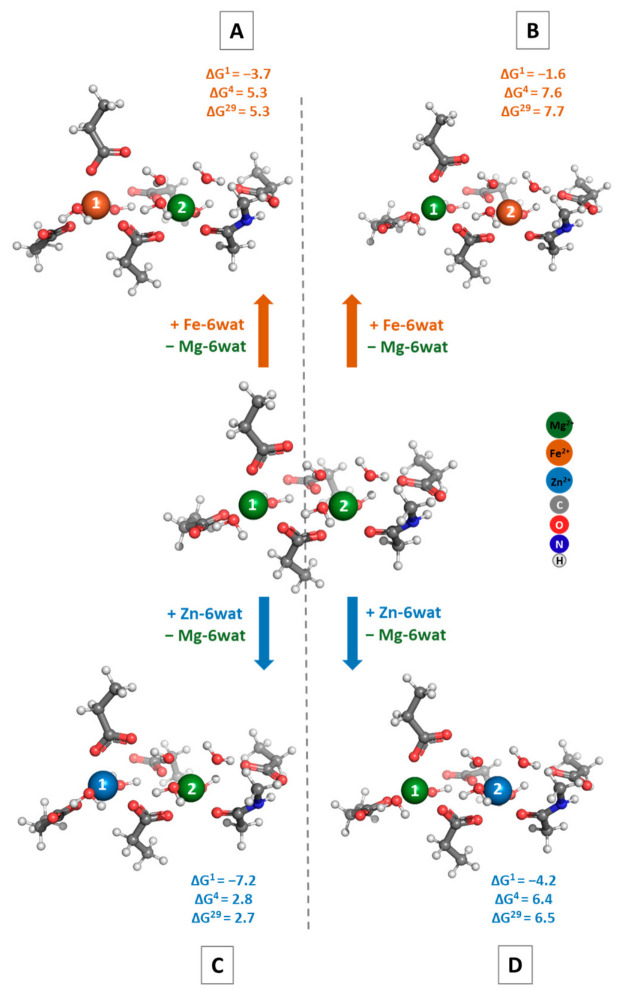
B3LYP/6-31+G(3d,p) fully optimized binuclear metal centers, containing Mg^2+^, Fe^2+^ and Zn^2+^ cations and Gibbs energies of Mg^2+^ → Fe^2+^/Zn^2+^ exchange (in kcal/mol): substitution of Mg_1_^2+^ (**A**) and Mg_2_^2+^ (**B**) cations with Fe^2+^; substitution of Mg_1_^2+^ (**C**) and Mg_2_^2+^ (**D**) cations with Zn^2+^.

Apart from Mn^2+^, PPM1A can be activated by Mg^2+^ cations as well [11,13,20]. What is the metal selectivity of the Mg-Mg binding site relative to that of its Mn-Mn counterpart? To answer this question, we modeled a Mg-Mg structure by substituting Mn^2+^ in the optimized Mn-Mn binding site with Mg^2+^, followed again by full geometry optimization (without any constraints) and frequency evaluations. Again, Fe^2+^ and Zn^2+^ were considered as contenders of the native Mg^2+^ cation. The optimized Mg-Mg structure is shown in Figure 3. The overall shape of the Mg-Mg center, as compared to that of the Mn-Mn construct, is preserved during the optimization. The former, however, is more compact that the latter: the mean Mg_1_^2+^ and Mg_2_^2+^–ligand bond distances shrink by 0.102 and 0.122 Å, respectively, relative to those in the Mn_1_-Mn_2_ active site. This suggests increased stability of the Mg-Mg metal center relative to its Mn-Mn analog and reflects on the gas phase Gibbs energies of the Mg^2+^ → Fe^2+^/Zn^2+^ exchange: ΔG^1^s, still negative, are generally higher (implying increased Mg^2+^ over → Fe^2+^/Zn^2+^ selectivity) than those of the Mn^2+^ → Fe^2+^/Zn^2+^ replacement (Figure 2) and vary between −1.6 and −3.7 kcal/mol (Mg^2+^ → Fe^2+^; Figure 4A,B) and −4.2 and −7.2 kcal/mol (Mg^2+^ → Zn^2+^; Figure 4C,D). Transferring the reacting entities from gas phase to condensed media reverses the trend and yields positive ΔG^4^s and ΔG^29^s (Figure 4A–D). The main reason for this change is the substantial difference in the solvation free energy of the Mg–aqua complex, on one side, and those of the Fe–aqua and Zn–aqua complexes, on the other: ΔG_solv_^78^(Mg-6wat) = −199.9 kcal/mol whereas ΔG_solv_^78^(Fe/Zn-6wat) = −209.0/−210.2 kcal/mol. Thus, the free energy gain associated with the release of the outgoing Mg-6wat species is overshadowed by the higher desolvation penalty of the incoming Fe/Zn-6wat complexes (R1). Unlike the Mn-Mn binding site, which appears to be vulnerable to a Fe^2+^/Zn^2+^ attack (negative ΔG^4^s and ΔG^29^s; Figure 2), the Mg-Mg construct seems to be well protected against Mg^2+^ → Fe^2+^/Zn^2+^ substitution (positive ΔG^4^s and ΔG^29^s; Figure 4).

These results are further supported by additional multilayer ONIOM calculations at the B3LYP/6-31+G(3d,p):UFF level of theory as implemented in the Gaussian 16 suite of programs [55] for reactions presented in Figure 4C,D by performing full geometry optimization of the PDB deposited structure of the binuclear center at 10 Å around the metal and calculating the surrounding amino acid residues with the aid of molecular mechanics without imposing any constraints. The outcome at the ONIOM level reproduces well the geometric characteristics of the binding sites, along with the general energetics trends. Table 3 provides a comparison of the mean metal–ligand distances between the two computational approaches.

The mean metal–ligand distances differ by about 0.01–0.02 Å depending on the computational approach. The arrangement of the ligands coincides as well, since the metal preserves its octahedral or penta-coordination. The only exception is the surrounding around the first magnesium ion in the Mg-Mg center which is octahedral at pure DFT and penta-coordinated at the multilayer ONIOM calculations. Note, however, that the migrating ligand is a flexible water molecule, and this outcome could be one of all possible local minimums.

### 3.3. Trinuclear Mg-Mg-Mg Binding Site

**Figure 5:**
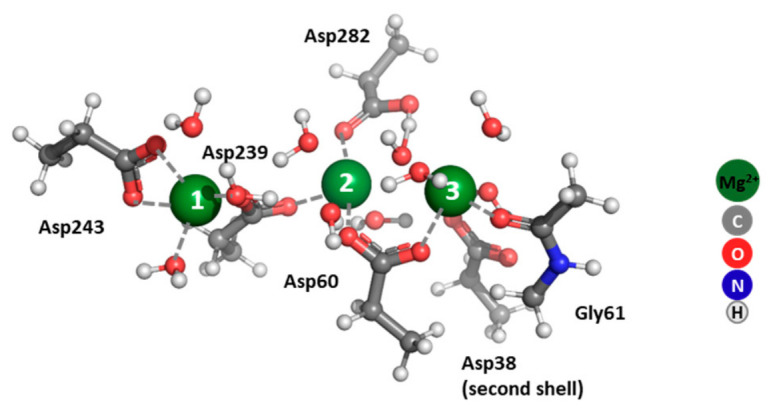
B3LYP/6-31+G(3d,p) fully optimized trinuclear Mg^2+^ binding site of PPM1A.

Tanoue et al. have found that the Mg^2+^-activated PPM1A may contain an additional third metal cation in the enzyme binding site [14]. Therefore, we were prompted to model and study the selectivity properties of this trinuclear active center. We modeled it after the 6B67 PDB structure [19] where the Ca^2+^ cations (used in the crystallization process) were substituted by Mg^2+^ cations in our study. The optimized structure of the Mg-Mg-Mg construct is shown in Figure 5. As seen, the three metal cations are bridged by aspartate residues: Asp239 connects Mg_1_^2+^ and Mg_2_^2+^, and Asp60 serves as a bridge between Mg_2_^2+^ and Mg_3_^2+^. Additionally, Mg_2_^2+^ and Mg_3_^2+^ are linked by an H_2_O/OH^−^ ligand. Non-bridging Asp282 binds Mg_2_^2+^ in a monodentate fashion whereas Asp243 coordinates Mg_1_^2+^ bidentately. The backbone carbonyl group of Gly61 is a part of the Mg_3_^2+^ first coordination layer.

**Figure 6:**
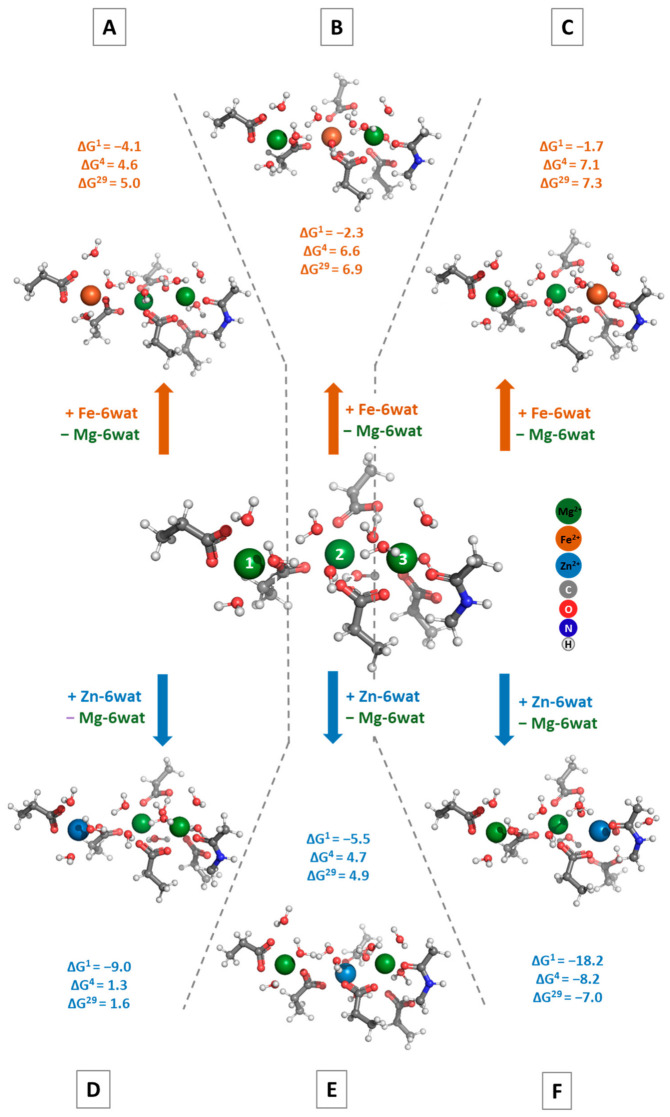
B3LYP/6-31+G(3d,p) fully optimized trinuclear metal centers, containing Mg^2+^, Fe^2+^ and Zn^2+^ cations and Gibbs energies of Mg^2+^ → Fe^2+^/Zn^2+^ exchange (in kcal/mol): substitution of Mg_1_^2+^ (**A**), Mg_2_^2+^ (**B**) and Mg_3_^2+^ (**C**) cations with Fe^2+^; substitution of Mg_1_^2+^ (**D**), Mg_2_^2+^ (**E**) and Mg_3_^2+^ (**F**) cations with Zn^2+^.

The competition between each of the three Mg^2+^ cations and non-native metal species (Fe^2+^ or Zn^2+^) was studied. Optimized structures of the mixed Mg^2+^–Fe^2+^/Zn^2+^ complexes and the respective Gibbs energies of the Mg^2+^ → Fe^2+^/Zn^2+^ exchange are presented in Figure 6. Displacing Mg^2+^ with Fe^2+^ follows a pattern, similar to that of the binuclear Mg-Mg construct (Figure 4): gas phase Gibbs energies for all reactions are negative, whereas those for the condensed phase evaluations turn positive. ΔG^4^s and ΔG^29^s in both cases fluctuate between ~5 and ~8 kcal/mol. The results obtained imply that the trinuclear binding site, similarly to its binuclear counterpart, can withstand attacks by ferrous cations in physiological conditions. The zinc cation, however, appears to be capable of breaching the “defense” system of the Mg-Mg-Mg binding site: as calculations suggest, displacing the native cation from binding site 3 by the intruder results in highly negative Gibbs energies of Mg^2+^ → Zn^2+^ substitution in both the gas phase and condensed media (Figure 6F). This is due to the change in the metal coordination pattern upon cation exchange: while Mg_3_^2+^ in the unsubstituted structure is hexacoordinated (Figure 5), in the Mg-Mg-Zn construct it is tetracoordinated (two water molecules had been relegated to the metal’s second coordination layer), which is a configuration highly favorable for the Zn^2+^ cation. Zinc at site 3 once again reached tetrahedral coordination during QM/MM optimization at the ONIOM B3LYP/6-31+G(3d,p):UFF level of theory as implemented in the Gaussian 16 suite of programs, providing evidence that the protein matrix does not hinder the transfer of the two water molecules to the second coordination shell of the metal. On the other side, Mg_1_^2+^ and Mg_2_^2+^ cannot be displaced by Zn^2+^ in condensed media (positive ΔG^4^s and ΔG^29^s in Figure 6D,E).

### 3.4. Trinuclear Mn-Mn-Mn Binding Site

**Figure 7:**
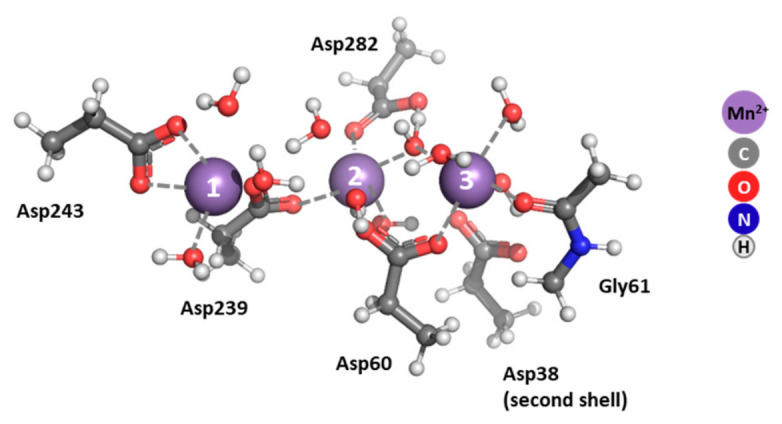
B3LYP/6-31+G(3d,p) fully optimized trinuclear Mn^2+^ binding site of PPM1A.

**Figure 8:**
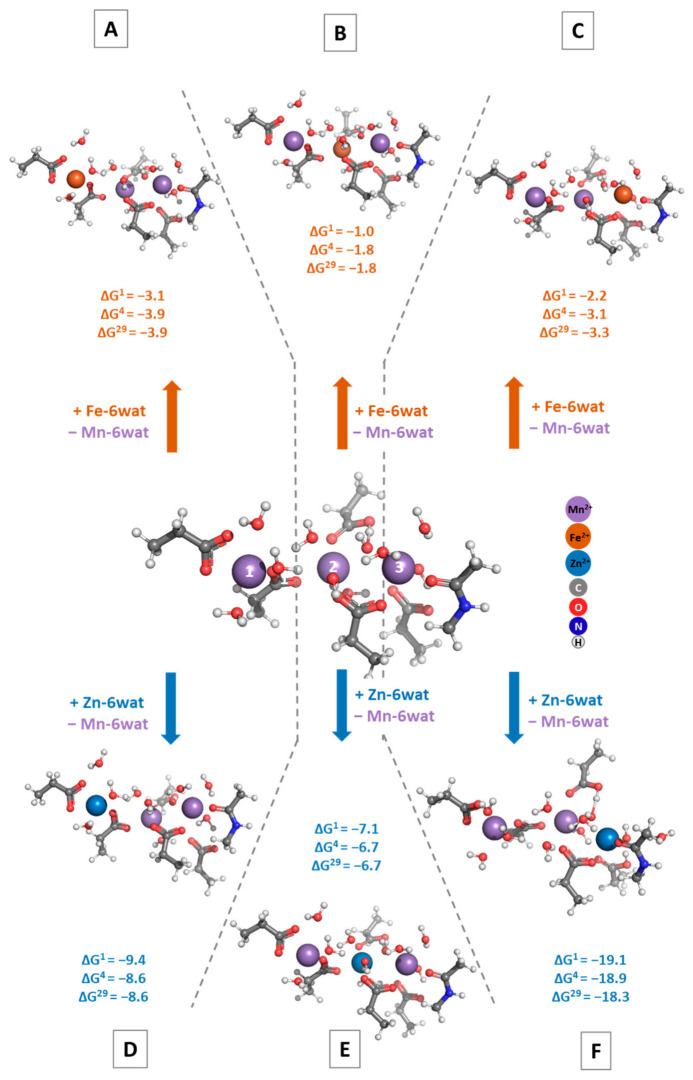
B3LYP/6-31+G(3d,p) fully optimized trinuclear metal centers, containing Mn^2+^, Fe^2+^ and Zn^2+^ cations and Gibbs energies of Mn^2+^ → Fe^2+^/Zn^2+^ exchange (in kcal/mol): substitution of Mn_1_^2+^ (**A**), Mn_2_^2+^ (**B**) and Mn_3_^2+^ (**C**) cations with Fe^2+^; substitution of Mn_1_^2+^ (**D**), Mn_2_^2+^ (**E**) and Mn_3_^2+^ (**F**) cations with Zn^2+^.

A trinuclear Mn-Mn-Mn binding site was modeled after the optimized Mg-Mg-Mg construct (Figure 5) where the Mg^2+^ cations were substituted with Mn^2+^ cations followed by full geometry optimization of the complex without imposing any constraints. The optimized Mn-Mn-Mn complex is shown in Figure 7. The overall structure of the new manganese construct appears similar to that of its magnesium counterpart as the two ions are characterized by an ionic radius of 0.72/0.83 Å for Mg^2+^/high-spin Mn^2+^ in their octahedral complexes, respectively [56]. Still, the average bond distances between the metal and ligating oxygen atom from the amino acid residues are about 0.1 Å longer in the manganese bound active center: the three Mn^2+^ stand at 2.15 Å from Gly61, 2.14 Å from Asp60, 2.23 Å from Asp282, 2.17/2.02 Å from Asp239 and at 2.19/2.17 Å from Asp243, whereas the three Mg^2+^ are positioned at 2.07 Å from Gly61, 2.05 Å from Asp60, 2.13 Å from Asp282, 2.09/1.94 Å from Asp239 and at 2.10/2.05 Å from Asp243. The metal–oxygen bond distances from the surrounding water molecules increase even more—by about 0.2 Å—in the manganese trinuclear construct as compared to the magnesium-bound one. Hence, the less compact Mn^2+^ bound center is more vulnerable to an outer attack since substituting each of the Mn^2+^ cations with Fe^2+^ is now thermodynamically favorable evidenced by all negative Gibbs energies of metal exchange in Figure 8. The calculations suggest that there is no clear preference of the attacking ferrous cation for the particular position of the displaced manganese cation (binding site 1, 2 or 3) as ΔGs for all three reactions fluctuate in very narrow limits (Figure 8A–C). Conversely, dislodging Mn_3_^2+^ from binding site 3 is the reaction of choice for the incoming Zn^2+^: ΔGs for the Mn^2+^ → Zn^2+^ substitution in the Mn-Mn-Zn construct (Figure 8F) are much lower (more favorable) than those for the respective Zn-Mn-Mn (Figure 8D) and Mn-Zn-Mn (Figure 8E) complexes. Again, as discussed in the previous paragraph, it is the transition from hexacoordinate Mn^2+^ in the Mn-Mn-Mn construct to tetracoordinate Zn^2+^ in the Mn-Mn-Zn complex that drives the reaction to strongly negative ΔGs. Generally, a Mn^2+^ → Zn^2+^ exchange in the trinuclear manganese complex is more favorable (lower Gibbs energies) than the Mn^2+^ → Fe^2+^ replacement.

## 4. Discussion

The obtained DFT results reveal substantial differences in the metal selectivity of the modeled structures mimicking the PPM1A active bi- and trinuclear centers depending on the nature of the native metal ions occupying the catalytic site. In particular, the Mn-containing active centers in all studied compositions appear significantly more susceptible to substitution by other biologically relevant divalent cations such as Fe^2+^ and Zn^2+^. The calculated Gibbs energies for the Mn^2+^ → Fe^2+^/Zn^2+^ exchange are consistently negative in both the gas phase and condensed media models for the Mn^2+^-bound systems, indicating thermodynamically favorable substitution processes. In contrast, the Mg^2+^-containing active sites generally exhibit considerably higher resistance toward metal exchange, suggesting a more robust and selective coordination environment. These observations indicate that the intrinsic physicochemical properties of the competing metal ions play a dominant role in governing metal selectivity when considering first and second shell binding residues of the active sites in PPM1A. Notably, experimental data provide evidence that PPM1A exhibits significant affinity for Mn^2+^ in vitro, but preferably binds Mg^2+^ in vivo [14]. One reason for that is the difference in the free metal concentrations in a living cell which is in the micromolar range for Mn^2+^ but at a higher, millimolar level for Mg^2+^. Our investigation sheds additional light on this from the thermodynamic point of view: Mg^2+^-loaded structures are predicted to be more robust and better protected against invasion by other metallic species (Figure 4 and Figure 6). On the other side, the calculations suggest that a modeled site replicating up to 10 Å the metal bound center is not well protected from a Zn^2+^ attack (Figure 2 and Figure 8). Indeed, as experimental findings reveal, Zn^2+^ (as well as Cd^2+^) inhibits the PPM1A enzymatic activity by replacing the cognate metal cation from the metal binding site [57]. This especially interesting result concerning the behavior of Zn^2+^ at binding site 3 of the Mg–Mg–Mg model and in all positions of the manganese trinuclear active center is once again demonstrated in the current study. The calculations suggest that Zn^2+^ can preferentially disrupt this site through a transition from a hexacoordinated to a tetracoordinated environment, consistent with the known preference of Zn^2+^ for lower coordination numbers and more directional bonding geometries. This local coordination rearrangement appears to provide an additional thermodynamic driving force for selective Zn^2+^ incorporation at this position. Such behavior may have important implications for understanding how non-native metal ions perturb the structural organization and catalytic competence in the vicinity of the phosphatase active sites.

The present calculations also suggest that Fe^2+^ substitution within the model of the PPM1A catalytic center is thermodynamically feasible. Although the obtained negative ΔG values do not directly demonstrate catalytic activation, the results are consistent with experimental observations indicating that Fe^2+^ may support PPM1A activity under certain conditions [13]. In addition, the optimized Fe-containing models preserve an active-site organization closely resembling that of the native systems in the area of the simulated region, further supporting the possibility that ferrous ions may occupy catalytically relevant coordination environments. We may speculate that sequestering Fe^2+^ from the surrounding milieu, in case of native metal deprivation, may appear beneficial for the PPM1A enzyme, which will still remain functional in these conditions.

In general, the relative stability trends closely follow the Irving–Williams series, reflecting the stronger complexation tendencies of divalent metal ions such as Zn^2+^ and Fe^2+^ compared to Mg^2+^ and Mn^2+^. At the same time, the solvation properties of the corresponding aqua complexes also contribute significantly to the overall thermodynamic balance of the exchange reactions. The calculations further indicate that factors such as the number of metal ions in the simulated region of the catalytic center and the solvent accessibility of the binding site appear to play a less pronounced role compared to the intrinsic coordination preferences of the metal ions themselves. Nevertheless, the present study employs simplified cluster-based static models focused on the metals’ first and second coordination spheres. Consequently, long-range electrostatic interactions, protein flexibility, solvent dynamics, and second-shell effects are not explicitly represented. These factors may influence the accessibility and stability of some of the observed coordination rearrangements in the native enzymatic environment. Despite these limitations, the employed DFT approach provides internally consistent comparative insight into the thermodynamic factors governing metal competition in PPM1A. Future investigations employing larger cluster models, QM/MM methodologies, and molecular dynamics simulations would be valuable for further exploring the contribution of the full protein environment and dynamic effects to metal selectivity and catalytic regulation in this important phosphatase system.

## 5. Conclusions

The presented study demonstrates that the metal selectivity of the models representing the PPM1A active site is fundamentally determined by the identity of the native metal ion. Specifically, constructs containing Mn^2+^—whether in binuclear or trinuclear configurations—are highly susceptible to substitution by other biogenic cations such as Fe^2+^ and Zn^2+^, as evidenced by consistently negative Gibbs energies for the Mn^2+^ → Fe^2+^/Zn^2+^ exchange in both gas phase and condensed media. In contrast, Mg^2+^-containing active sites (with the exception of the Mg-Mg-Zn construct) exhibit much greater resistance to such substitutions, with positive Gibbs energies indicating thermodynamic protection against ferrous and zinc attack under physiological conditions.

The principal determinants of this metal competition in the modeled constructs are the intrinsic properties of the metal cations—particularly their position in the Irving–Williams series—and the solvation characteristics of their aqua complexes. Factors such as the number of metal ions in the catalytic center and the degree of solvent exposure play a comparatively minor role in dictating selectivity. Notably, the computational findings are consistent with a number of experimental observations: magnesium preferentially binds in the active site of PPM1A (more robust Mg^2+^–bound site is much better protected against an outer attack); zinc (as well as cadmium) ions block the PPM1A enzyme by substituting the native ion (negative ∆G for the Zn^2+^ → Mn^2+^ exchange in the binuclear center), and non-native metals like Fe^2+^ can activate PPM1A (although negative ΔG values for Fe^2+^ displacement indicate thermodynamic feasibility of metal substitution, but do not by themselves demonstrate catalytic activation of PPM1A, the highly similar and preserved geometry of the Fe^2+^/Mn^2+^ binuclear complexes partially implies that Fe^2+^ may contribute to PPM1A activity under certain conditions).

Overall, these results provide atomistic-level insight into the regulatory factors that govern metal ion selectivity in two models of the active centers of PPM1A, highlighting the superior stability and fidelity conferred by Mg^2+^ over Mn^2+^. This understanding not only advances our knowledge of metalloprotein chemistry but also has potential implications for therapeutic strategies targeting PPM1A in various disease contexts. Future studies employing complementary computational techniques, such as QM/MM and molecular dynamics simulations, could further enrich our understanding of metal ion selectivity in this and related systems.

## Figures and Tables

**Table 3:** Comparison between structures obtained at different levels of theory.

	Mean M-O Distance, Å		
Construct		DFT: B3LYP/6-31+G(3d,p)	QM/MM (ONIOM): B3LYP/6-31+G(3d,p):UFF
Mg-Mg	Mg_1_–O	2.11 (octahedral)	2.09 (pentacoord.)
	Mg_2_–O	2.05 (pentacoord.)	2.03 (pentacoord.)
Mg-Zn	Mg_1_–O	2.05 (pentacoord.)	2.04 (pentacoord.)
	Zn-O	2.14 (octahedral)	2.13 (octahedral)
Zn-Mg	Zn-O	2.08 (pentacoord.)	2.09 (pentacoord.)
	Mg_2_–O	2.1(1) (octahedral)	2.1(0) (octahedral)

## Data Availability

The original contributions presented in this study are included in the article. Further inquiries can be directed to the corresponding author.
